# Forest fire detection system using wireless sensor networks and machine learning

**DOI:** 10.1038/s41598-021-03882-9

**Published:** 2022-01-07

**Authors:** Udaya Dampage, Lumini Bandaranayake, Ridma Wanasinghe, Kishanga Kottahachchi, Bathiya Jayasanka

**Affiliations:** Department of Electrical, Electronic and Telecommunication Engineering, Faculty of Engineering, General Sir John Kotelawala Defence University, Ratmalana, 10390 Sri Lanka

**Keywords:** Electrical and electronic engineering, Ecology

## Abstract

Forest fires have become a major threat around the world, causing many negative impacts on human habitats and forest ecosystems. Climatic changes and the greenhouse effect are some of the consequences of such destruction. Interestingly, a higher percentage of forest fires occur due to human activities. Therefore, to minimize the destruction caused by forest fires, there is a need to detect forest fires at their initial stage. This paper proposes a system and methodology that can be used to detect forest fires at the initial stage using a wireless sensor network. Furthermore, to acquire more accurate fire detection, a machine learning regression model is proposed. Because of the primary power supply provided by rechargeable batteries with a secondary solar power supply, a solution is readily implementable as a standalone system for prolonged periods. Moreover, in-depth attention is given to sensor node design and node placement requirements in harsh forest environments and to minimize the damage and harmful effects caused by wild animals, weather conditions, etc. to the system. Numerous trials conducted in real tropical forest sites found that the proposed system is effective in alerting forest fires with lower latency than the existing systems.

## Introduction

Forest fires are disasters that cause extensive damage to the entire world in economic, ecological, and environmental ways. These fires can be caused by natural reasons, such as high temperatures that can create spontaneous combustion of dry fuel such as sawdust, leaves, lightning, etc., or by human activities, such as unextinguished campfires, arson, inappropriately burned debris, etc^1^. According to research, 90% of the world’s forest fire incidents have occurred as a result of the abovementioned human carelessness^1^. The increase in carbon dioxide levels in the atmosphere due to forest fires contributes to the greenhouse effect and climate change. Additionally, ash destroys much of the nutrients in the soil and can cause erosion, which may result in floods and landslides.

At earlier times, forest fires were detected using watchtowers, which were not efficient because they were based on human observations. In recent history and even the present day, several forest fire detection methods have been implemented, such as watchtowers, satellite image processing methods, optical sensors, and digital camera-based methods^2^, although there are many drawbacks, such as inefficiency, power consumption, latency, accuracy and implementation costs. To address these drawbacks, a forest fire detection system using wireless sensor networks is proposed in this paper.

Wireless sensor networks (WSNs) are self-configured and infrastructure-free wireless networks that help monitor physical or environmental conditions and pass these data through the network to a designated location or sink where the data can be observed and analyzed^3^. Efficiency and low power consumption are the major advantages of a WSN. In the proposed detection system, wireless sensor nodes are deployed according to cellular architecture to cover the entire area with sensors to monitor temperature, relative humidity, light intensity level, and carbon monoxide (CO) level using a microcontroller, transceiver module, and power components. The power supply to the sensor node is provided using batteries as the primary power supply, and solar panels are used as the secondary power supply. These sensor nodes are specially designed with a spherical shape to withstand damage caused by environmental conditions as well as animals.

The sensor readings for each parameter are checked with a preset threshold ratio and a ratio that is calculated continuously in the node in real time, and only the ratios that exceed the preset ratio are sent from the sensor node to the base station for further analytical processing. The network utilized for this transmission is in the architecture of tree topology considering facts such as low power consumption, reduced latency, less complexity, etc. Cluster heads are used in this network to gather data from several sensor nodes and pass them on to the base station or the gateway node. The gateway node is an interface that connects the network with the secondary analysis process.

For the analysis process, a machine learning regression model was used along with threshold ratio analysis to enhance detection accuracy. For the training and testing process of the model, data were collected during the fire and no fire situations in different areas and under different climatic conditions. During the data collection process, 7000 data samples were collected, where a data sample included temperature, relative humidity, light intensity level, and CO level at a particular time. Eighty percent of the collected data were randomly used as training data for the model, and the remaining 20% were used as test data.

If the outcome of the machine learning model indicates a fire in a specific area, a text message will be sent to the mobile phone numbers of the authorized officers in responsible units. As this process is designed with a minimum delay, the fire can be detected within the initial stage, and the responsible parties can take necessary actions in a shorter period, which will minimize the damage.

### Related work

Forest fire detection has been a focus of many researchers for the last decade because of increased forest fire case reports from all over the world due to severe damage to society and the environment. Many methods have been proposed to detect forest fires, such as camera-based systems, WSN-based systems, and machine learning application-based systems, with both positive and negative aspects and performance figures of detection. Due to the higher probability of accurate and early detection due to the use of multiple sensor sources and deployment of sensor nodes in areas not visible to satellites, wireless sensor networks have a more positive outlook, and they have become the more applicable technology in many fields^4^.

Many researchers have focused on environmental parameters, such as air temperature, relative humidity, barometric pressure, sound, light intensity, soil moisture, and wind speed and direction, along with gases, such as CO, CO_2_, methane, H_2_, and hydrocarbons apart from smoke, to detect forest fire conditions by considering the variations in these parameters during a fire^5,6^, and sensors have been selected according to the range, sensitivity, power consumption, and cost^7,8^.

As supplying power to a sensor node is a challenging task in forested areas, utilizing only battery options is difficult because they do not last long, and distributing power using a wire would require a higher cost to deploy in a large forest. Therefore, many researchers have proposed solar-powered systems as secondary power sources along with rechargeable batteries as the main power source^4,6^, while some researchers have proposed solar batteries because they last longer^9^. To reduce the power consumption of sensor nodes, techniques such as keeping selected components active while others are deactivated have been proposed^10–12^.

Most WSN-based detection systems are centered around a base station due to the memory and processing limitations of the nodes. Important and partially processed data are transferred to the base station through wireless media for processing and enabling relevant actions, while the base station also acts as the gateway between the sensor nodes and the system user^4,9^.

When constructing a WSN, communicating data among the relevant entities is the main objective, and star topology and mesh topology-based networks have been proposed in many papers because of the different attributes in their systems. A mesh topology was chosen over a star topology because of its ability to self-organize, self-configure, and automatically establish among nodes in a network^13^. As a smaller number of nodes involved for transmission results in minimum energy consumption, concepts based on cluster heads have been used^14^. To minimize the loss of energy and data packages during transfer, a cluster-tree network topology structure was proposed^15^. Considering the sensing range of a node, fault tolerance, and energy consumption, a paper has proposed applying the on-demand k-coverage technique that provides event detection using static nodes with variable sensing ranges. This technique utilizes the maximum detection performance with the minimum power consumption for an event^16^. A survey on rare event detection has mentioned many event detection strategies that deliver maximized detection capability, minimized detection delay and low energy consumption, such as duty cycle, component deactivation, overpopulation/node redundancy, collaboration, and energy harvesting^17^.

To reduce deployment cost and power consumption, a paper proposes a novel localization scheme that divides the whole forest area into different grids and allocates them to respective zones with another 8 neighboring grids. One centroid node from those grids, which is called the initiator node, predicts whether the zone is highly active (HA), medium active (MA), and low active (LA). Here, HA zones send data continuously to the base node through the interior node, MA zones send data periodically, and LA zones do not transfer data in the status that manages power consumption effectively^18^. Another author proposed obtaining data from the sensors every 2 min if there is the potential of a forest fire or obtaining data every 15 min otherwise to reduce the energy wastage^19^.

To place sensor nodes in the most effective configuration to detect fire conditions, a sensor node was proposed at three different heights to perform different parameter measurements, while some authors have suggested covering sensor nodes to avoid direct sunlight exposure and minimize the false alarm rate^4,10^. As the network connectivity of service providers in forest areas is not robust, communication techniques that use dedicated network paths such as LoRa, ZigBee, and XBee have been used as the communication infrastructure. When considering attributes such as transmission range, high security, low power consumption (LPWAN protocol), and other relevant configurations, most papers have suggested using the LoRa module for transmission^13,19^.

Most papers have suggested having threshold value-based fire detection on a sensor node, and if the exceeded threshold has remained the same, then a sink determines the location and will send an alarm to the fire department^11^. Because of the environmental parameter variations according to the place and time, threshold values are configured by the user considering geographic situations, climatic changes, seasonal changes, etc. after sensors obtain the data from the surrounding^10^.

A fusion information process was proposed, where information from multiple sources is considered in making the final decision, which is better than using those sources individually, and two algorithms based on the threshold ratio method and Dempster-Shafer theory were used^4^. To enhance the detection accuracy, machine learning applications have been proposed in many papers based on different machine learning approaches, such as support vector machine (SVM) classification^14^ and regression techniques, such as logistic regression. However, applying machine learning techniques to fire detection systems has many limitations, such as the limited amount of energy, the energy required for data processing, the short range of communication and limited computations, the complexity of ML algorithms when executing on sensor nodes, and the difficulty of being distributed on every sensor node^20,21^.

## Materials and methods

### Sensor node design

**Figure 1:**
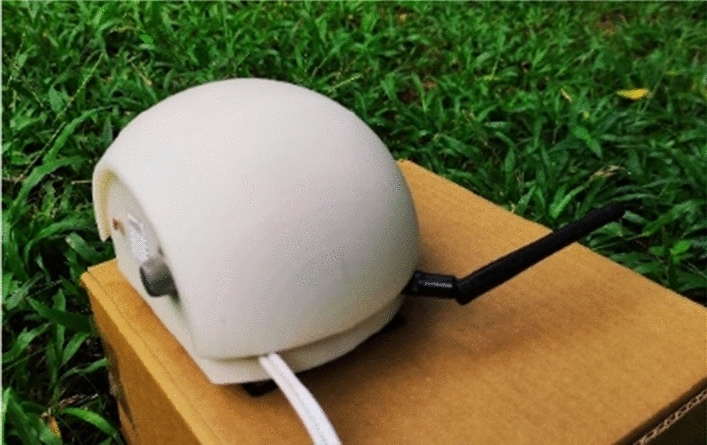
Spherical external design.

**Figure 2:**
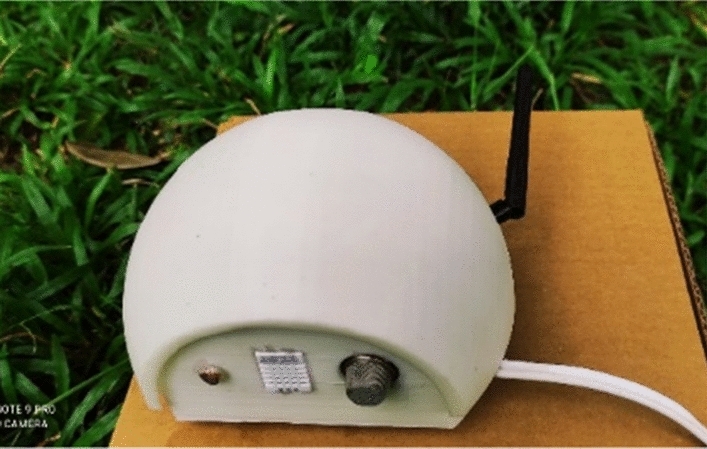
Sensor place at the base.

**Figure 3:**
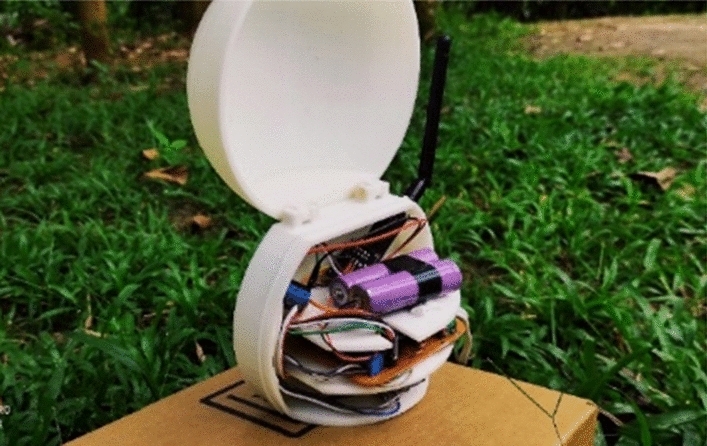
Inertial component placing.

**Figure 4:**
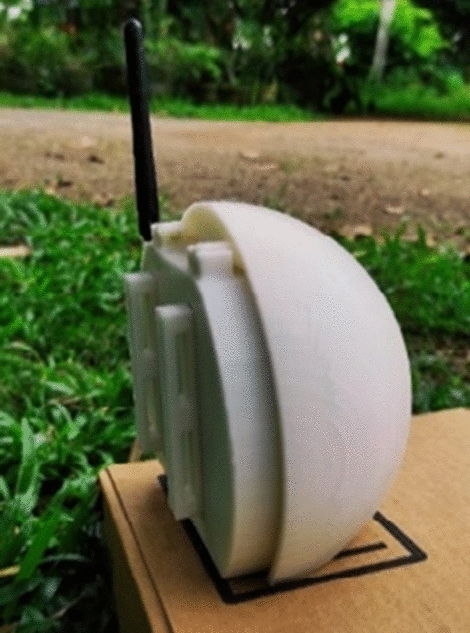
Mounting supporters.

The sensor node is designed with a spherical shape to withstand external forces and with characteristics to prevent damage due to the harsh conditions prevailing in tropical forests (Fig. 1). The sensor node is used to detect the temperature, humidity, light intensity level, and CO level, which are detected from the lower side of the node facing downwards (Fig. 2). The interior of the node is designed in three layers to place the sensors and modules (Fig. 3). The topmost layer is designed to place a lithium-ion battery, while the middle layer is designed to place the microcontroller, voltage regulator, and connecting board. The lowest layer, which is also the base of the node, is used to fix the sensors that are used to monitor the environmental conditions mentioned above. All the sensors are fixed facing downwards to protect them from harmful effects of environmental conditions such as rain, strong winds, and objects such as leaves. From the side of the node, a hole is designed to take the antenna of the transceiver outside. The mounting brackets of the node on the tree trunk and supporters are connected on the rear side of the sensor node (Fig. 4).

Here, to monitor temperature and relative humidity, the DHT22 sensor is used, while the LDR sensor and MQ9 sensor are used to monitor light intensity and CO level, respectively. An Arduino Nano board is selected as the microcontroller device, which is small in size and flexible with a wide variety of applications. The nrf24L01 module is used as the transceiver on every sensor node, cluster head, and base node to communicate with each other. Rechargeable lithium iron cells (2 cells in series, 2 cells in parallel) are used as the primary power source because of the improved battery life and low cost. Each 18650-lithium ion cell provides 3*:*7 V with a 4800 mAh capacity. As the secondary power supply, the solar panel is used, and it gives 5 W power and 12 V voltage output.

### Sensor node deployment

After conducting many tests, the maximum sensing range of a sensor node is determined to be five meters. After conducting a site survey of the average foliage height, the sensor node is mounted on a tree trunk one meter above the ground to conduct detection tests of initial stage surface fire conditions effectively. The deployment of the sensor nodes in a forest is carried out after a similar site survey of the average foliage height and as per the cellular architecture (Fig. 7) to cover the whole area of interest. As the maximum sensing range of a sensor node is five meters, a sensor node can cover a five-meter radius.

**Figure 5:**
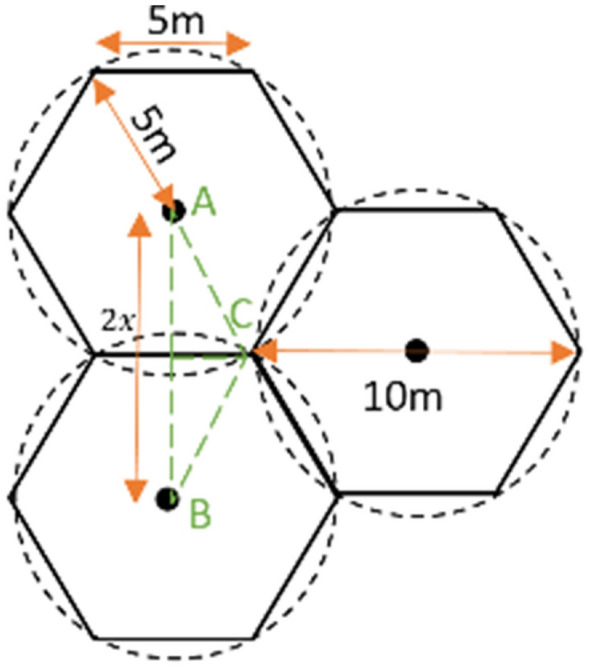
Sensor node deployment.

**Figure 6:**
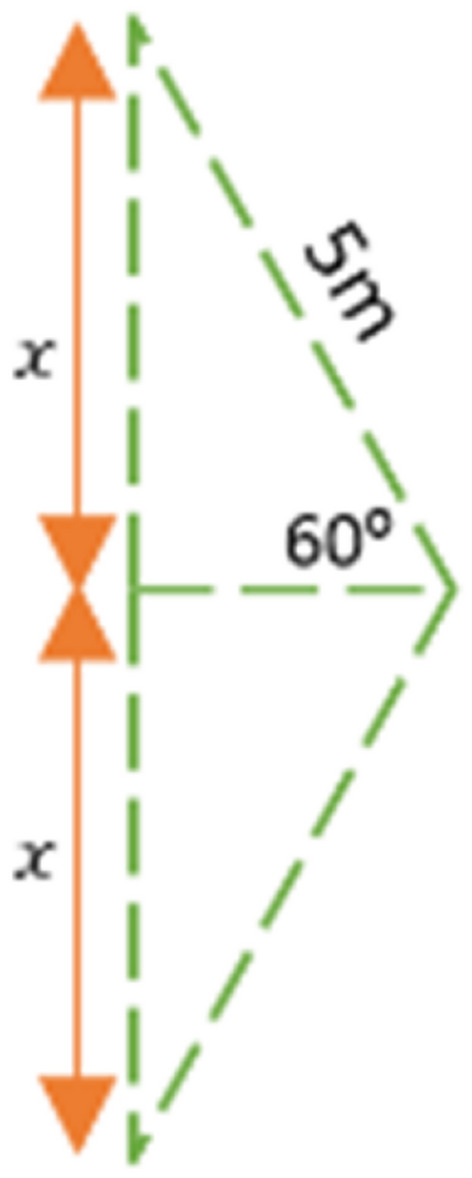
Distance between two sensor nodes.

According to Figs. 5 and 6, the distance between two sensor nodes is calculated as follows.

$$x = 5m \times \sin \left( {60} \right) = 4.33m$$

$$2x = 4.33m \times 2 = 8.66m$$

**Figure 7:**
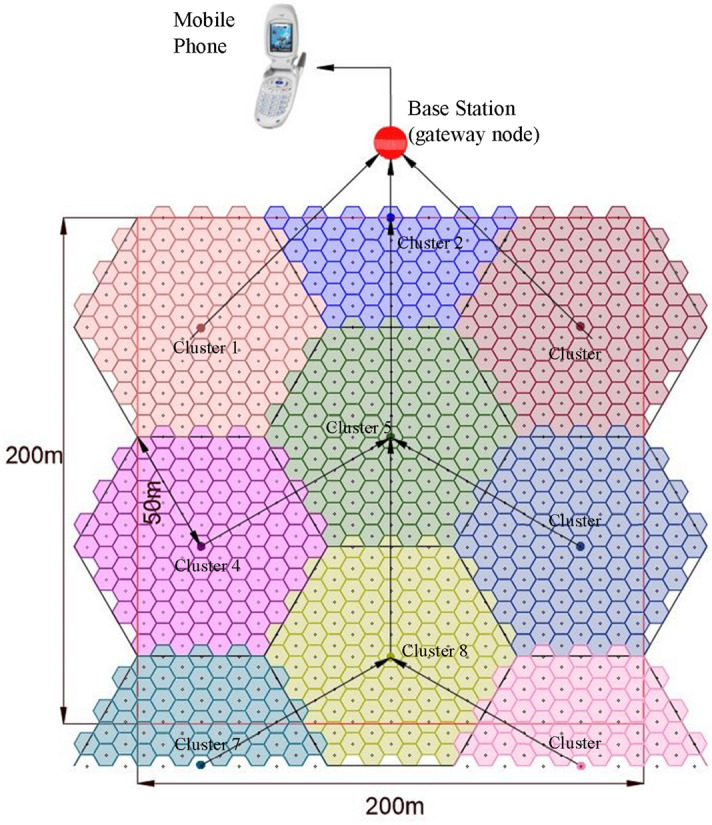
Sensor Nodes and Cluster Heads Deployment for 200 × 200 m area.

For the data transmission from the sensor node to the gateway node (Fig. 7), a nrf24L01 module is utilized, which has a maximum transmission range of 100 m in a forest-type environment. To collect data from sensor nodes beyond 100 m and for ease of communication, the sensor nodes are arranged into clusters. Each cluster has a cluster head that collects data from sensor nodes and transmits them to the gateway node. Deployment of the cluster heads is also arranged according to the cellular architecture, where a single cluster head covers an area of a circle with a 50 m radius when considering the maximum range of the transceiver module. As per the calculations, the distance between two cluster heads is 86.66 m, and each cluster head collects data from 100 sensor nodes. The set of data values sent by the sensor nodes on the condition of exceeding the threshold ratio are collected by the relevant cluster heads and passed to the gateway for further analysis. A cluster head deployed beyond 100 m from the gateway node can pass data through intermediate cluster heads.

Each cluster head is equipped with a lithium-ion battery, solar panel, Arduino nano module, and nrf24L01 module. In the base station, domestic power is used as the main power supply to the system, and it consists of a machine (PC) for processing purposes, an Arduino Nano module, a nrf24L01 module, and a SIM800 L module.

## Results and discussion

### Data collection

For the detection of fire conditions, two analytical methods are used, namely, threshold ratio analysis and analysis using a machine learning algorithm. To carry out these analytical processes, data were collected by creating several controlled fire conditions. The aforementioned conditions were created in an area of 1 m^2^, and the sensor node was mounted on a post one meter above the ground and placed one meter from the fire. The data collection was carried out in different climatic zones during the morning, afternoon, and night hours to capture the natural environmental variations throughout the whole year.

### Threshold ratio analysis

The environmental parameters, including temperature, relative humidity, light intensity level, and CO level, are monitored by the system under different climatic conditions in different climatic zones during the morning, afternoon, and night hours. The threshold ratio $R_{TH}$ is determined during these extensive trials. A ratio is calculated continuously within the sensor node by reading each parameter $R$ by the respective sensors with thirty-second periods. If the calculated ratio of a single parameter exceeds the threshold ratio value three consecutive times, then a set of ten data values are sent to the gateway node from each parameter. To determine the threshold ratio, data are collected at different areas and different times of the day.

$$Threshold\, Ratio = \frac{Current\, data\, value}{{Data\, value\, before\, 30s }}$$

1 $$Threshold\, Ratio \left( {R_{TH} } \right) = \frac{{d_{t} }}{{d_{t - 30} }}$$

**Figure 8:**
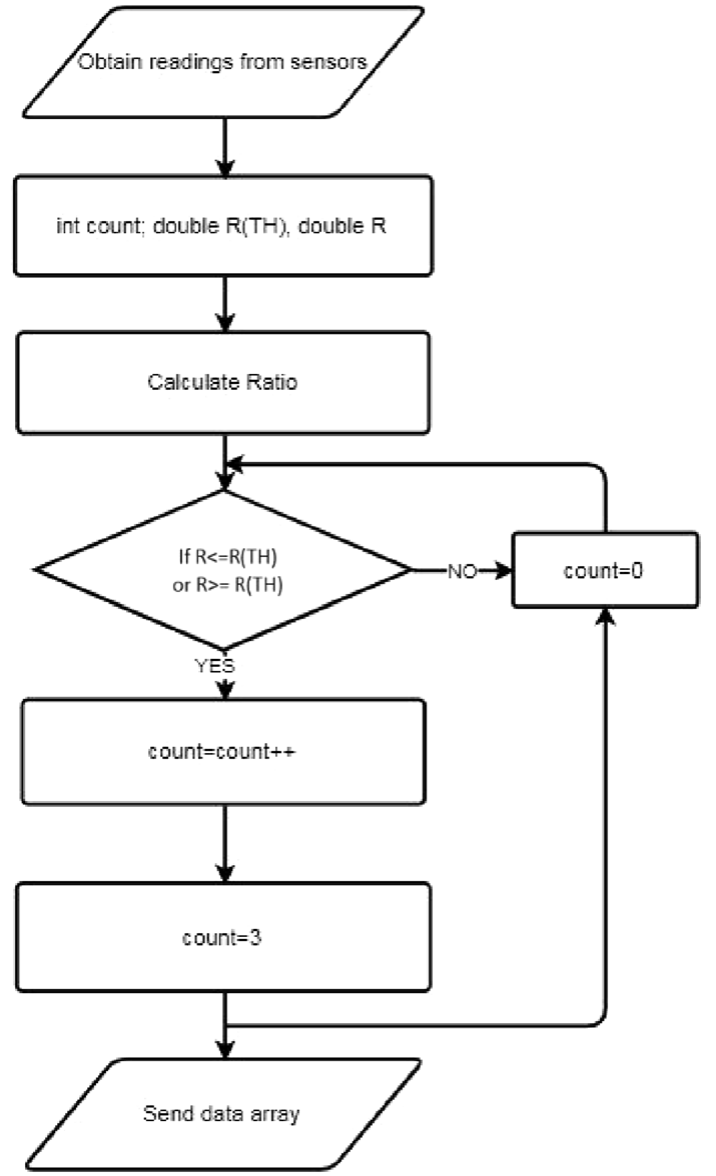
The decision flow of the sensor node.

The decision flow of the sensor node is depicted in Fig. 8. When the temperature and light intensity are checked, if the condition is $R\left( {TH} \right) < = R$, in regard to the humidity and CO level, the condition should be $R\left( {TH} \right) > = R$. Here the threshold ratio is denoted by $R\left( {TH} \right)$_._

### Machine learning algorithm

To minimize the effect of spontaneous or random errors and erroneous sensor readings, long-term pattern analysis is carried out by a machine learning algorithm.

The machine learning application is based on multiple linear regression techniques, which give the most accurate results in the case of building a relationship between multiple independent variables and dependent variables.

**Figure 9:**
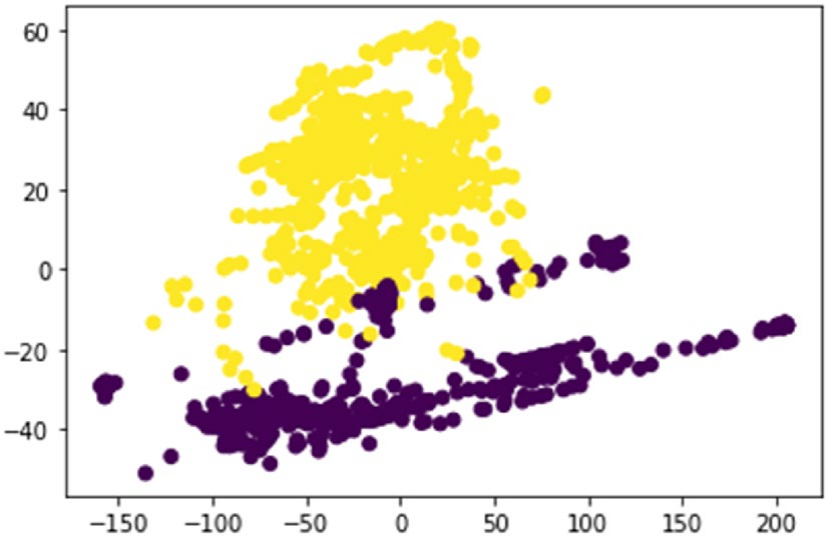
Clustered data samples using K-means method.

A dataset of 7000 samples was collected where a single data sample comprised values of temperature, relative humidity, light intensity level, and CO level at a particular instance within a certain climatic zone. These data samples were collected by monitoring no-fire situations as well as controlled fire situations that were created in the different climatic zones during the morning, afternoon, and night hours to capture the natural environmental variations throughout the whole year. The K-means technique was used to separate the data samples into two clusters: fire and no fire, as plotted in Fig. 9.

Eighty percent of the prepared dataset is used for the training process of the model, and the remaining 20% is used for the testing process. The dataset was trained by using a multiple linear regression model.

The data sent from sensor nodes after threshold analysis are collected at the gateway node and fed into the machine learning model. If a fire situation is detected after an analysis, the model provides an output as a fire situation along with the area where it has occurred. Then, using a SIM800 L module (quad-band GSM/GPRS module) placed on the base station, the output will be sent to the responsible authorities as a text message.

### Field testing

The initial testing of the system was performed at land adjoining the Kanneliya Forest Reserve [6.183120723327043, 80.4165345965101], Sri Lanka, and the other was at land adjoining the Knuckles Mountain Range Wildlife Reserve [7.45857419273649, 80.77990606784306], Sri Lanka, which are often subjected to frequent forest fires. A controlled fire was created, and sensor nodes were placed to detect the fire. One cluster head and the base node were implemented for the transmission of data and further analysis. In both locations, the tests were performed in the morning, afternoon, and night to check the system applicability to different times of the day.

### Results

For performance analysis of the sensor node, the predefined threshold ratio values were determined for each parameter, namely, temperature, relative humidity, light intensity level, and carbon monoxide level. To determine the threshold ratio values for all four parameters, data values were obtained by creating 15 controlled fires, and values were determined for fire situations created at different climatic zones during the morning, afternoon, and night hours. The graphs of Figs. 6, 7, 8,9 show each parameter’s ratio *vs* time, where the ratio was obtained by calculating the ratio between the current reading and the reading before 30 s as per Eq. (1). The readings that were used to plot the graphs below commenced 60 s before the time instant when the fire was started.

**Figure 10:**
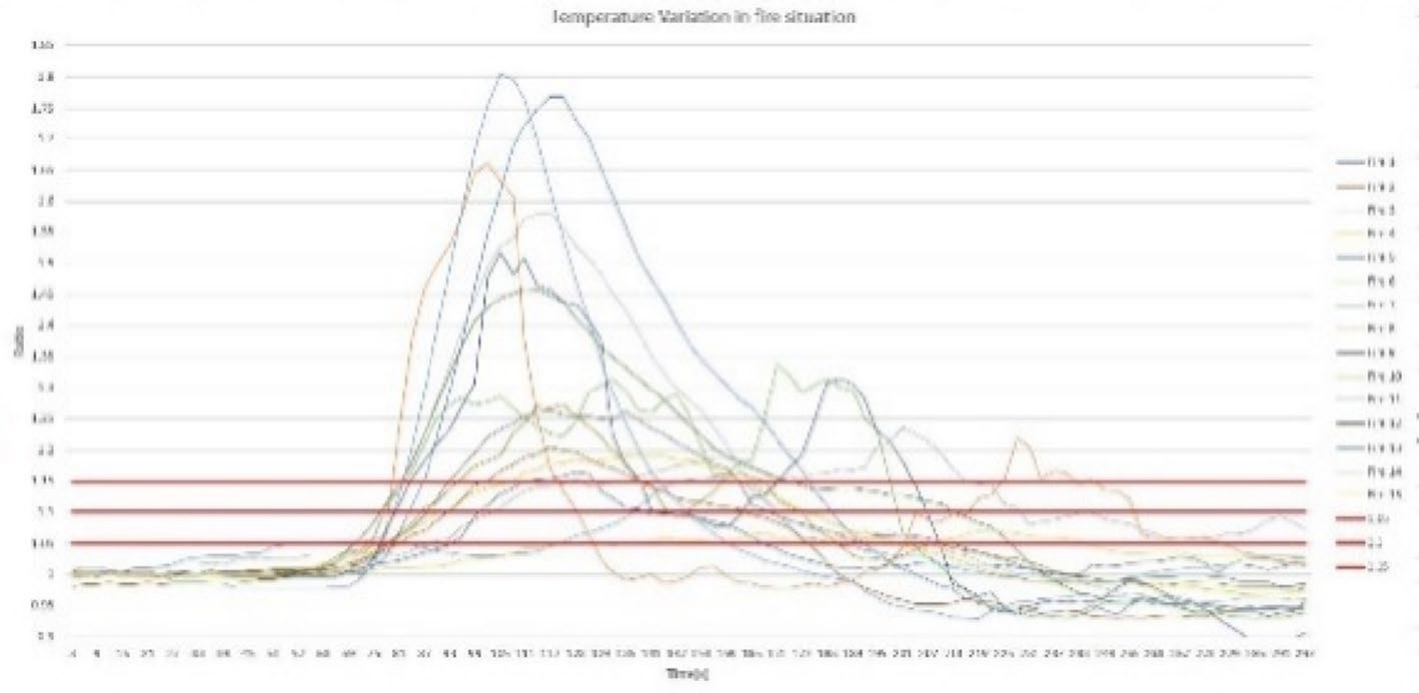
Temperature ratio vs Time.

**Figure 11:**
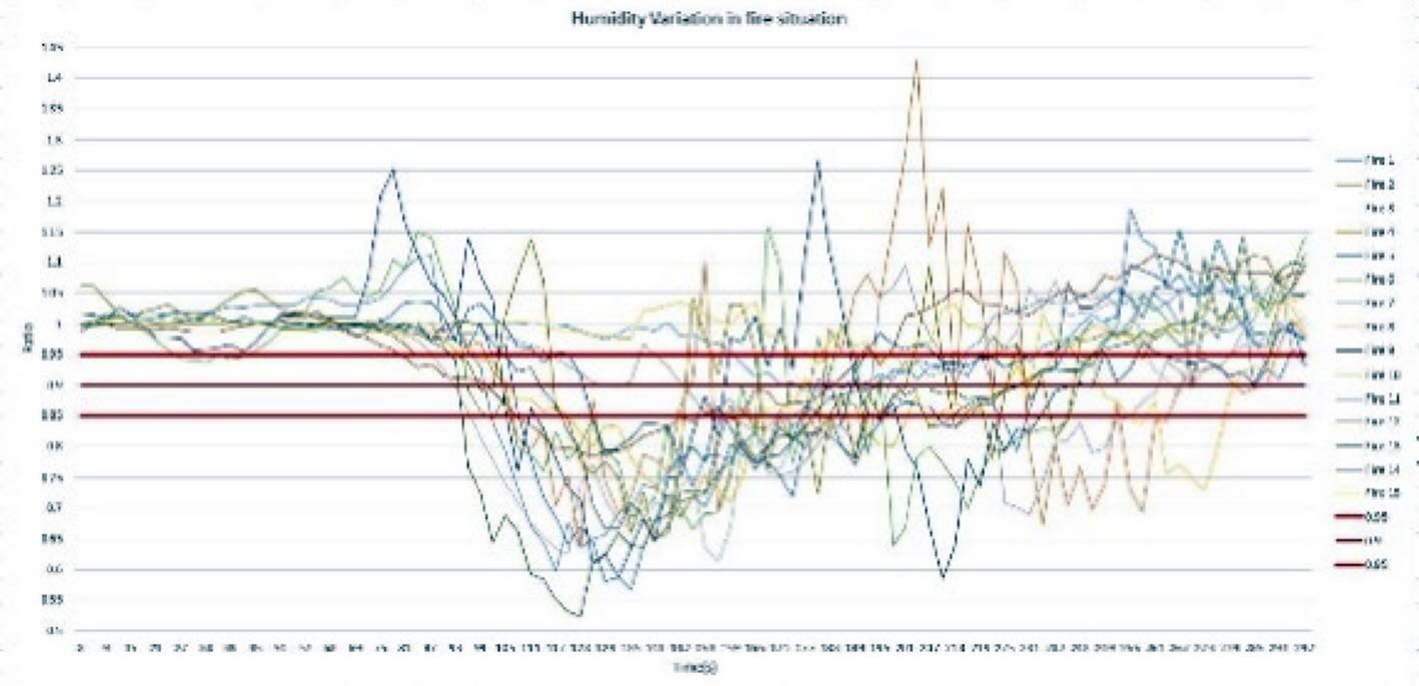
Humidity ratio vs time.

**Figure 12:**
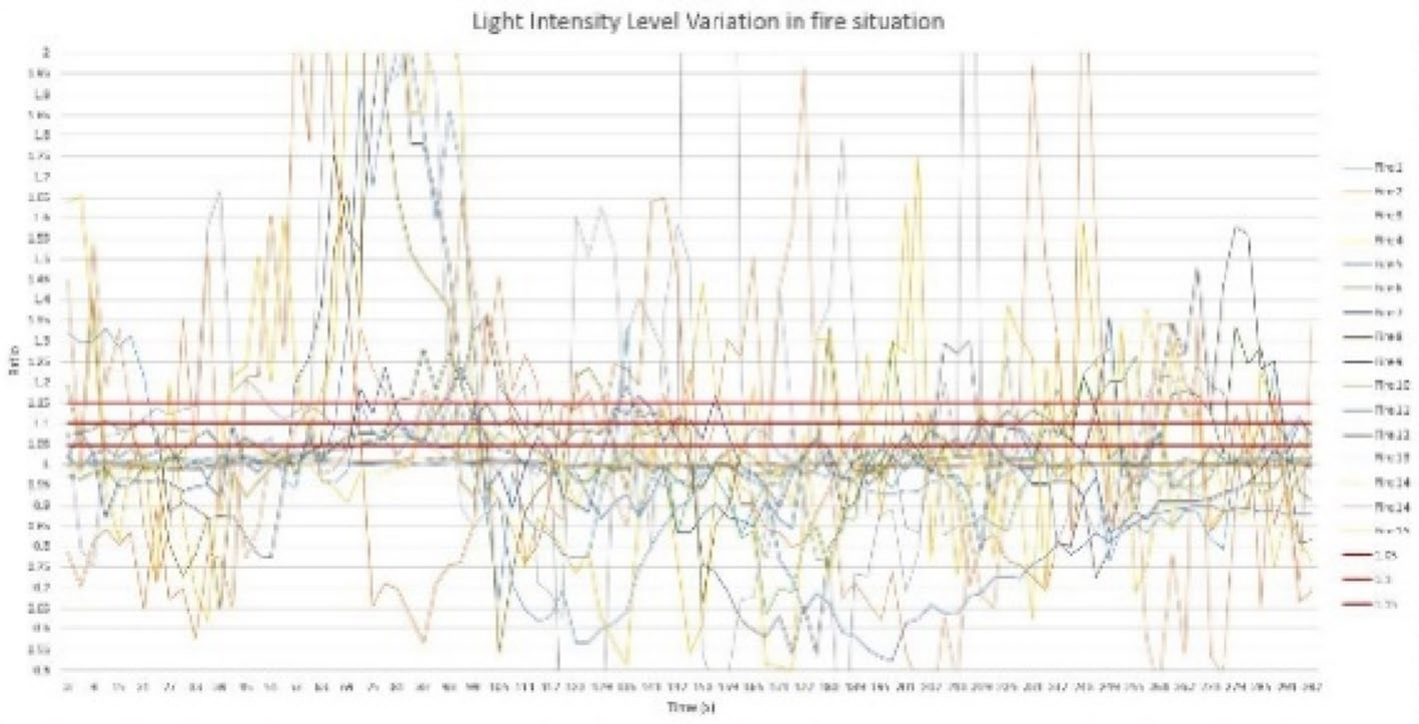
Light intensity ratio vs time.

**Figure 13:**
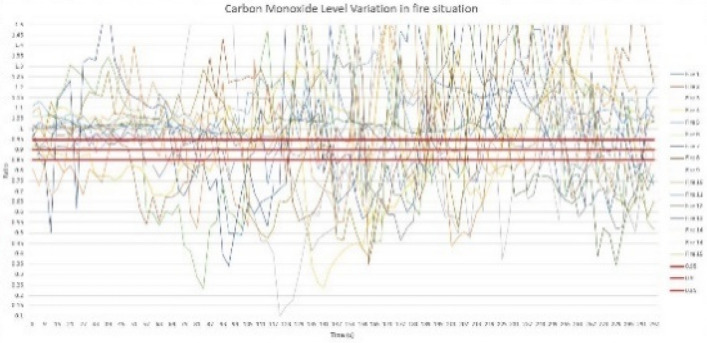
CO level ratio vs time.

Three possible threshold ratios were selected for all four parameters considering the variation profiles of each parameter, which was plotted using 15 fire situations, and each fire situation was analyzed with the selected threshold ratio values. For temperature and light intensity, 1.15, 1.1, and 1.05 were selected, as those parameter ratios should be higher than 1, while for humidity and CO levels, 0.85, 0.8, and 0.95 were selected, as those ratios should be less than 1 in fire situations. When considering the temperature ratio plot depicted in Fig. 10, the lowest ratio value that reaches almost every fire situation, 1.05, was decided as the threshold ratio value for temperature after careful observation of the plot; otherwise, some fire situations might not be detected by the system. In regard to the threshold ratio of relative humidity presented in Fig. 11, the highest value, 0.95, which captures almost all 15 fire situations, was considered. Referencing Fig. 12, the light intensity level extensively varies during a fire and could show a significant variation even in no fire situations. Considering 1.05 and 1.1 as the threshold ratio would detect normal situations as fire situations and result in false alarms. Therefore, the threshold ratio value for the light intensity level is set to 1.15. As the readings of CO levels from the MQ9 sensor are within a narrow range of values, as observed in Fig. 13, a small change in the reading may show a considerable drop-off ratio even in a no-fire situation. Therefore, 0.95 and 0.9 would trigger false alarms. Therefore, the threshold ratio value for the CO level is set to 0.85. The selected threshold ratio values are presented in Table 1.

**Table 1:** Decided threshold ratio value for each parameter.

Parameter	Threshold ratio value
Temperature	1.05
Relative humidity	0.95
Light intensity level	1.15
CO level	0.85

The sensor node was designed in a spherical shape to be stable and damage resistant. When designing the node, the sensors and other equipment placement in the node extensively consider mounting ability and safety, as depicted in Figs. 1, 2, 3, 4.

**Figure 14:**
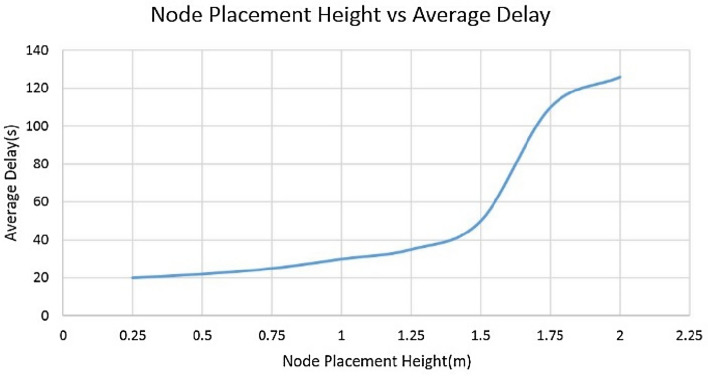
Node placement height vs average delay.

After performing many tests, the 5 m range was observed as the effective area that a single sensor node can cover. The variation in average delay *vs* height was observed, as depicted in Fig. 14; hence, the height of the mounting point from the ground level was determined to be 1 m.

**Figure 15:**
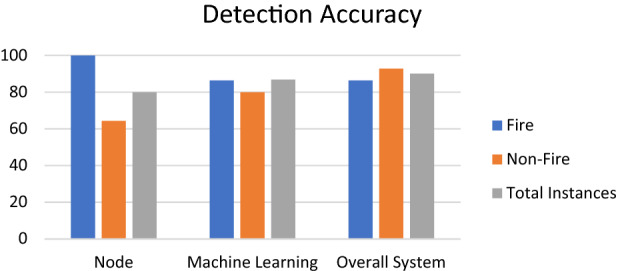
Accuracy graph of system, node and machine learning model.

On training the machine learning regression model using 80% of the dataset with 7000 samples and testing it by the remaining 20%, the theoretical accuracy of the final output obtained was 81%. After testing the system for twenty-two fire scenarios and twenty-eight no-fire scenarios, node accuracy, practical accuracy of the machine learning model, and overall system accuracy (considering all fire and no fire situations) were calculated, and the data are presented in Table 2 and Fig. 15.

**Table 2:** Accuracy graph of system, node and machine learning model.

Scenario	Total instances	Accurate instances	Erroneous instances	Accuracy rate (%)	Error rate (%)
**Node**					
Fire	22	22	0	100	0
No FIRE	28	18	10	64.28	35.72
Total instances	50	40	10	80	20
**ML**					
Fire	22	19	3	86.36	13.64
No fire	10	8	2	80.00	20.00
Total instances	38	33	5	86.84	13.15
**Overall system**					
Fire	22	19	3	86.36	13.64
No fire	28	26	2	92.85	7.15
Total instances	50	45	5	90	10

A statistical t-test was performed to determine the significance of the parameters that were utilized to detect fire conditions. The test was conducted for all four parameters: temperature, relative humidity, light intensity level, and carbon monoxide level considering fire and no fire situations. The probability values obtained for all four parameters were less than the critical value ($\propto$ = 0.05), as indicated in Table 3. In advance, the probability values were less than 0.001, which implies that those parameters have a strong correlation with the condition of fire. Therefore, it can be concluded that the above parameters can be well utilized for the detection of fire conditions^22^.

**Table 3:** Significance level for each parameter.

Parameter	Significance level (*p*-value)
Temperature	1.77 × 10^−44^
Relative humidity	1.03 × 10^−45^
Light intensity level	1.73 × 10^−6^
CO level	8.16 × 10^−12^

## Conclusion

The proposed system for forest fire detection using wireless sensor networks and machine learning was found to be an effective method for fire detection in forests that provides more accurate results. Here, to obtain a more accurate outcome within the lowest latency, the analysis takes place within both the sensor node and at the base station. For the system, to fit any weather condition, climatic condition, or area, a threshold ratio is introduced for analysis within the sensor node. In the case of node deployment, it can be mounted at any place in the forest even if there is no preinstalled network connectivity, as the transceiver module is based on dedicated built-in network infrastructure. Because of the primary power supply provided by rechargeable batteries with a secondary solar power supply, a solution is readily implementable as a standalone system for prolonged periods. The proposed system incorporated with the communication infrastructure alerted the relevant authorities with lower latency than the existing systems during the numerous test trials conducted in real tropical forest sites.

## Data Availability

The datasets generated and/or analyzed during the current study are available from the corresponding author on reasonable request.
